# Reliability of the Marlowe-Crowne social desirability scale in Ethiopia, Kenya, Mozambique, and Uganda

**DOI:** 10.1186/1471-2288-11-162

**Published:** 2011-12-02

**Authors:** Alexander Vu, Nhan Tran, Kiemanh Pham, Saifuddin Ahmed

**Affiliations:** 1 International Emergency and Public Health Fellowship Program, Department of Emergency Medicine, The Johns Hopkins School of Medicine, Baltimore, USA; 2 Health Systems Program, Department of International Health, The Johns Hopkins Bloomberg School of Public Health, Baltimore, USA; 3 Department of Population & Family Health and Department of Biostatistics, The Johns Hopkins Bloomberg School of Public Health, Baltimore, USA

## Abstract

**Background:**

Studies of HIV often use self-reported surveys to measure sexual knowledge, attitudes, and practices. However, the self-reported data are vulnerable to social desirability (SD), a propensity of individuals to report favorable responses. The Marlowe-Crowne Social Desirability Scale (MC-SDS) was developed as a measure of the effect of social desirability, but it has not been adapted for or used in Africa. This study aimed to apply the MC-SDS nested in an HIV behavioral intervention program and to measure its reliability in four African countries.

**Methods:**

The MC-SDS was adapted based on consultations with local stakeholders and pilot tested in Ethiopia, Kenya, Mozambique, and Uganda. Trained interviewers administered the modified 28-item MC-SDS survey to 455 men and women (ages 15-24 years). The scores for the social desirability scales were calculated for all participants. An analysis of the internal consistency of responses was conducted using the Cronbach's α coefficient. Acceptable internal consistency was defined as an α coefficient of ≥ 0.70.

**Results:**

Mean social desirability scores ranged from a low of 15.7 in Kenya to a high of 20.6 in Mozambique. The mean score was 17.5 for Uganda and 20.6 for Mozambique. The Cronbach's α coefficients were 0.63 in Kenya, 0.66 in Mozambique, 0.70 in Uganda, and 0.80 in Ethiopia.

**Conclusions:**

The MC-SDS can be effectively adapted and implemented in sub-Saharan Africa. The reliability of responses in these settings suggest that the MC-SDS could be a useful tool for capturing potential SD in surveys of HIV related risk behaviors.

## Background

The United States committed $18.8 billion between 2003 to 2008 to the President's Emergency Plan for AIDS Relief (PEPFAR) [1]. In its original formulation, more than $3 billion was allocated for the prevention of HIV [1]. The majority of these investments are evaluated through the use of self-reported surveys of HIV knowledge, attitudes, and sexual practices (KAP) to assess a population's understanding about and behavior towards HIV [2].

A pervasive concern with self-reported surveys used to evaluate these programs is how the collected data is affected by social desirability [3]. Social desirability is a tendency of respondents to reply in a manner that would be viewed positively by their social peers or that are consistent with social norms and expectations [4]. It has been found that when answering questions concerning sensitive topics, such as questions about sexual behaviors, it is common for respondents to describe their behaviors in a more favorable manner [5]. This effect of SD may reflect a deliberate misrepresention of their true behavior. Alternatively, SD may reflect individuals' need to seek approval from their peers, and as such, respondents may provide favorable responses in order to gain approval from their peers. Regardless of the reason, the possibility that respondents misrepresent socially undesirable behavior is concerning to researchers as it puts into question the validity of self-reported survey data. The effect on self-reports remains the same: individuals who are highly affected by SD will score higher on measures of "agreeableness" and other socially desirable traits [6].

Moreover, cultural norms and attitudes regarding sexual practices differ greatly across countries and cultures. The tendency for the effect of SD to be observed in self-reported sexual practices and behaviors varies according to the setting in which the surveys are implemented [7]. The measure of the SD score of the individual should be considered in the context of and compared to the mean scores of the overall target population. It is only when the SD score of the individual differs significantly from the overall mean scores within the population under study that there is a concern for SD affecting the individual's responses to sensitive questions [6].

Marlowe and Crowne proposed and developed a social desireability scale (MC-SDS) to measure socially desirable responses [8]. The original MC-SDS contains 33 strong true or false statements that influence the individual to respond in a manner that conforms to social expectations regarding behaviors, attitudes, and beliefs. Using this scale, the effect of SD can be observed in individuals who have tendencies to seek approval from their social peers or who may misrepresent their true behavior so as to make a good impression. These individuals would tend to score high on the social desirability scale.

The effect of SD can potentially be taken into account when interpreting the results of self-reported surveys [8]. Since the original scale was released in 1960, MC-SDS has been tested in diverse settings and has been shown to be a reliable instrument to measure SD [9,10]. The scale has also been applied in multi-ethnic populations: the United States [11], India [12], the Netherlands [13], and the Philippines [14]. However, the MC-SDS has not been used in sub-Saharan Africa, and thus its reliability is unknown in this region.

PEPFAR identified fifteen priority countries at its inception, thirteen of which are in Africa [1]. In these priority countries, PEPFAR funded a range of prevention activities that aimed to increase awareness of HIV and related risk behaviors as well as to promote abstinence and fidelity. The evaluation of these efforts have largely relied on either self-administered or interviewer-administered KAP surveys. To the best of our knowledge, the potential effect of SD on the results of HIV KAP surveys in the African context is poorly understood and has not been adequately addressed. The overall aim of this study is to apply and measure the reliability of the MC-SDS in four African countries: Ethiopia, Kenya, Mozambique, and Uganda. We accomplished this aim by: 1) adapting and pilot testing the MC-SDS in sub-Saharan Africa; 2) measuring the internal consistency of the responses to the MC-SDS in these four countries; and 3) exploring opportunities for the application of the MC-SDS as a tool for assessing the potential impact of social desireability on self-reported surveys of HIV related risk factors in sub-Saharan Africa.

## Methods

### Formative Research

This study was initiated in July 2008 with consultations with local NGOs, community leaders working on HIV prevention programs, and other stakeholders in each of the four countries to determine the feasibility of and plan for the implementation of the study. The results of this early work provided insights into current practices related to the use of self-reports to monitor sexual practices as well as the nature of the challenges that are experienced with this form of survey research.

In addition, a comprehensive review of the literature on HIV-related survey research in Africa and the effect of SD on self-reported surveys was performed. The literature review identified several scales and techniques that were developed to assess the effect of social desirability on self-reported data and to correct for this problem (the SD scale developed by Edwards [15], the Lie scale from the Eysenck Personality Inventory [16], the bogus pipeline technique developed by Jones and Sigall [17], and the SD scale developed by Marlowe and Crowne [8] are a few examples). The decision to use the Marlowe Crowne SD scale for this study was based on the finding that the MC-SDS is one of the most commonly referenced and used measures of social desirability. The formative work also contributed to the identification of strategies for the pilot testing of the MC-SDS tool as well as the location and potential study populations for the study.

The study was reviewed and approved by the Johns Hopkins University School of Medicine Institutional Review Board for compliance with the University's policies on ethical research [18].

### Description, Adaptation, and Development of MC-SDS versions

The original MC-SDS questionnaire [8] consisted of 33 statements to which respondents are asked to answer "true" or "false" (Table 1). The tool was translated by local translators; back translations were then reviewed by the research team. Additional edits were made through an iterative process to ensure that the translations captured the essence of the statements. The statements were translated into local languages and then pilot tested with convenience samples to determine the level of understanding. This was carried out by the local research assistants under the supervision of investigators at Johns Hopkins University (JHU). The results of the pilot study were reviewed jointly by the JHU investigators and local research team, and final edits were made based on the feedback of the interviewers on the capacity of the general population to understand and respond to the statements in the questionnaire.

**Table 1:** Revised Marlowe-Crowne Social Desirability Scale

Original Elements of Marlowe-Crowne Social Desirability Scale	
1.	Before voting I thoroughly investigate the qualifications of all the candidates.
2.	I never hesitate to go out of my way to help someone in trouble.
3.	It is sometimes hard for me to go on with my work if I am not encouraged.
4.	I have never intensely disliked anyone.
5.	On occasion I have had doubts about my ability to succeed in life.
6.	I sometimes feel resentful when I don't get my way.
7.	I am always careful about my manner of dress.
8.	My table manners at home are as good as when I eat out in a restaurant.
9.	If I could get into a movie without paying and be sure I was not seen I would probably do it.
10.	On a few occasions, I have given up doing something because I thought too little of my ability.
11.	I like to gossip at times.
12.	There have been times when I felt like rebelling against people in authority even though I knew they were right.
13.	No matter who I'm talking to, I'm always a good listener.
14.	I can remember "playing sick" to get out of something.
15.	There have been occasions when I took advantage of someone.
16.	I'm always willing to admit it when I make a mistake.
17.	I always try to practice what I preach.
18.	I don't find it particularly difficult to get along with loud mouthed, obnoxious people.
19.	I sometimes try to get even rather than forgive and forget.
20.	When I don't know something I don't at all mind admitting it.
21.	I am always courteous, even to people who are disagreeable.
22.	At times I have really insisted on having things my own way.
23.	There have been occasions when I felt like smashing things.
24.	I would never think of letting someone else be punished for my wrongdoings.
25.	I never resent being asked to return a favor.
26.	I have never been irked when people expressed ideas very different from my own.
27.	I never make a long trip without checking the safety of my car.
28.	There have been times when I was quite jealous of the good fortune of others.
29.	I have almost never felt the urge to tell someone off.
30.	I am sometimes irritated by people who ask favors of me.
31.	I have never felt that I was punished without cause.
32.	I sometimes think when people have a misfortune they only got what they deserved.
33.	I have never deliberately said something that hurt someone's feelings.
	
**Question items removed from the final SDS version**	
1.	Before voting I thoroughly investigate the qualifications of all the candidates.
8.	My table manners at home are as good as when I eat out in a restaurant.
9.	If I could get into a movie without paying and be sure I was not seen I would probably do it.
27.	I never make a long trip without checking the safety of my car.
29.	I have almost never felt the urge to tell someone off.

As the questions were initially designed for use in the United States, some statements referenced cultural phenomena that were not relevant to low- and middle-income countries. This was revealed after the initial piloting of the translated MC-SDS questionnaire; in particular five statements were deemed inappropriate for use in the African context. For example, statement 29, which states "I have almost never felt the urge to tell someone off," was removed because the concept of "telling someone off" could not be translated in many of the local languages and it was believed that the essence of this statement was already captured in other statements. We omitted such statements from the original list of 33, resulting in a questionnaire with 28 statements. The statements removed from the original tool are shown in Table 1.

In all countries, the modified 28-item MC-SDS was translated from English to the official language, and where necessary, additional translations were done for local languages spoken in each of study catchment areas. In order to facilitate comparisons across the four countries, the same version of the survey that was developed and pilot tested in Ethiopia was used in the other three countries.

### Sample and Sampling Framework

This study was implemented in the four countries in conjunction with the evaluation of a PEPFAR-funded HIV prevention initiative called the Mobilization, Equipping, and Training (MET) Project implemented by an NGO, Samaritan's Purse. The goal of the MET Project was to reduce the incidence of HIV infection through behavioral modification, with an emphasis on abstinence and fidelity using a grassroots, community mobilization approach. The evaluation of the MET Program was conducted through surveys of HIV knowledge, attitudes towards people living with HIV, self-reports of sexual behaviors (such as abstinence, fidelity, and sex with commercial sex workers), as well as other HIV-related risk factors. In order to maximize use of the existing program infrastructure, the study was carried out in conjunction with the routine data collection activities already being conducted as part of the monitoring and evaluation of the MET Project.

Lot Quality Assurance Sampling (LQAS) was used as the sampling method for the KAP survey of the MET Project and for this study [19]. Respondents were identified using a stratified, three-stage probability sampling procedure. First, each program site where the MET program was operating was divided into 6 supervisory areas. Next, within each supervisory area, nineteen villages were selected based on the population-proportional-to-size method. Within each village, a sample of never-married respondents ages of 15-24 was selected randomly. Using LQAS, the total number of individuals sampled for the study was 455 (Table 2).

**Table 2:** Demographic Characteristics of the Study Population

Characteristics	Ethiopia	Kenya	Mozambique	Uganda
Total (n)	114	113	114	114

Gender (%)				
Male	48	52	50	50
Female	52	47	50	50

Age (%)				
15-19	81	51	72	75
20-24	19	48	28	25

Education (%)				
None	0	4	3	0
Primary	43	63	59	67
Secondary	48	32	39	33

### Data Collection & Analysis

**Figure 1:**
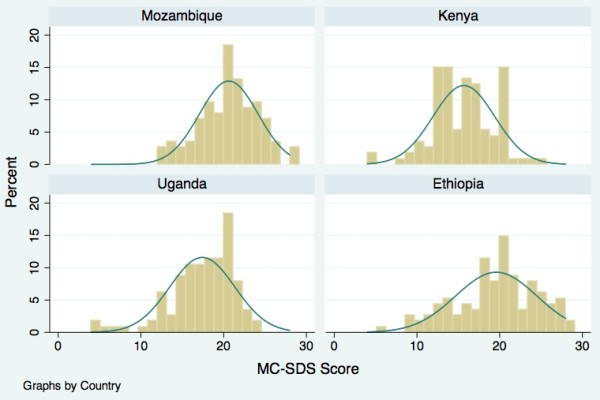
**MC-SDS Score by Country**. Distribution of MC-SDS Scores.

Ethiopia was the first study site; data collection commenced in November 2008 following the initial pilot testing. Data collection was then carried out in Kenya in April 2009, in Mozambique in May 2009, and in Uganda in July 2009. Data collectors in each site received a two-day training on the modified 28-item MC-SDS and survey research methodology. The questionnaire was administered by data collectors through face-to-face interviews. Data were entered into an Epi Info^® ^database and analyzed with STATA/SE^® ^version 11. Using the guidelines developed by Crowne and Marlowe, total MC-SDS scores were generated. Mean and median SD score were analyzed for each study population. A comparison of mean SD scores is shown in Figure 1 and Table 3.

**Table 3:** Mean MC-SDS^1 ^Scores (95% CI), by selected characteristics

Characteristics	Ethiopia	Kenya	Mozambique	Uganda
Overall	19.5 (18.6-20.4)	15.7 (15.0-16.4)	20.6 (19.9-21.2)	17.5 (16.7-18.2)

Gender				
Male	19.5 (18.2-20.8)	15.9 (14.9-16.9)	20.8 (19.8-21.7)	17.2 (16.2-18.3)
Female	19.7 (18.3-20.9)	15.4 (14.4-16.4)	20.5 (19.5-21.5)	17.6 (16.5-18.7)

Age				
15-19	19.8 (18.8-20.8)	15.8 (14.8-16.8)	20.9 (20.2-21.7)	17.2 (16.3-18.1)
20-24	18.8 (16.5-21.2)	15.5 (14.6-16.5)	19.9 (18.5-21.3)	18.1 (17.0-19.1)

^1^Scores are from the modified, 28-item version of the MC-SDS as described in the paper

To assess the internal consistency of the responses to the scale, the Cronbach's α coefficient was estimated for each target group in each of the four countries. The Cronbach's α coefficient, originally used to assess the consistency of psychometric test scores, provides an indication of the reliability of the instrument for question items that assess similar constructs [20]. Analyses using the Kuder-Richardson formula (K-20), developed specifically to test the reliability of dichotomous scales (true/false questions), were also carried out, and the results were almost identical to those obtained using the Cronbach's α. The respective α coefficient and KR-20 for each country are: 0.80 and 0.79 for Ethiopia; 0.63 and 0.63 for Kenya; 0.66 and 0.66 for Mozambique; and 0.70 and 0.70 for Uganda. We defined the threshold of acceptable internal consistency or reliability with an α coefficient of 0.70 or greater.

## Results

A total of 455 respondents participated in the study with a nearly even distribution of participants of both sexes in each country (Table 2). The overall distribution of females ranged from 48% to 52%. The majority of the participants were 15-19 years of age and had some form of education. In Ethiopia, a large proportion of the respondents (48%) had secondary schooling. In the other countries, most respondents had only a primary level education. These demographic characteristics are summarized in Table 2.

Figure 1 shows the distribution of the MC-SDS scores in the four countries. In Ethiopia, the MC-SDS scores ranged from 6 to 28, with a mean of 19.5. In Kenya, the MC-SDS scores ranged from 5 to 25, with a mean of 15.7, which was the lowest among the four country sites. The MC-SDS scores in Mozambique ranged from 12 to 28 with a mean of 20.6, the highest of the four countries. In Uganda, the MC-SDS scores ranged from 4 to 24 with a mean of 17.5. There were no significant differences of the MC-SDS mean scores between males and females or between the younger and older never-married respondent groups (Table 3).

Table 4 shows the Cronbach's α coefficients according to selected characteristics. It ranged from a low of 0.63 in Kenya to 0.66 in Mozambique, 0.70 in Uganda, and to a high of 0.80 in Ethiopia. In Ethiopia, there were no significant differences between males and females; individuals aged 15-19 years in Ethiopia had a slightly lower α score than individuals aged 20-24 years. In Mozambique there were no differences in the α score between male and females. The Cronbach's α coefficient was consistent across all groups in Uganda. In Kenya, which had the lowest overall α score, there were more marked differences between males and females and between the younger and the older age groups.

**Table 4:** Reliability Coefficient (Cronbach's α) of MC-SDS, by selected characteristics

Characteristics	Ethiopia	Kenya	Mozambique	Uganda
Overall	0.80	0.63	0.66	0.70

Gender				
Male	0.81	0.64	0.68	0.68
Female	0.81	0.72	0.68	0.70

Age				
15-19	0.79	0.71	0.65	0.72
20-24	0.85	0.58	0.71	0.72

## Discussion

The present study aimed to assess the reliability of the Marlowe-Crowne social desirability scale in Ethiopia, Kenya, Mozambique, and Uganda. The overall results show the MC-SDS to be a reliable instrument to assess for the effect of social desirabilty in the Africa. In Kenya and Mozambique, where the reliability coefficents were only marginally lower than the generally accepted threshold for adequate internal consistency of 0.70, the MC-SDS could still be considered a reasonably good tool with acceptable reliability. There were no significant differences in the mean SD scores among gender and age sub-groups. It was anticipated that age and gender may influence the degree to which individuals are willing to disclose personal information as this is often what is observed during surveys which solicit information about risk behaviors. However, these characteristics did not appear to impact the social desirability score in this region.

A major concern in implementing the MC-SDS outside of the United States is that the cultural context of the scale is not understood in the same way in other settings, thereby limiting the utility of the scale. However, as our results have demonstrated, with some minor adaptation and translation, the tool generates results that are reliable in diverse settings. The adequate reliability of responses to the MC-SDS in 2 of the 4 countries (Ethiopia and Uganda) supports its application in studies where sensitive information relating to sexual practices are solictited in self-reports. The MC-SDS could be used in conjunction with surveys used for monitoring of HIV/AIDS prevention programs which often rely on self-reports of abstinence and fidelity to measure program impact. The use of the MC-SDS can provide additional information regarding the potential effect of SD on individuals who participate in the self-reported surveys and thus help in the interpretation of the data, particularly in communities where self-reports of abstinence, fidelity, and condom use are very high.

Although the MC-SDS generated reliable responses in all four countries, there were differences in both the level of reliability as well as in the SD scores among the four countries. Several factors may account for the differences in internal consistency. First, there are clearly cultural differences and norms around self-disclosure as well as in the degree of pressure to conform to social norms and values among the four countries. It is possible that in Ethiopia, there is greater pressure to conform to social values. This resulted in the individual's propensity to respond more favorably to sensitive questions (as reflected by the high MC-SDS scores).

Moreover, the MC-SDS was designed as an instrument to measure specific social constructs in an American population. The lower levels of internal consistency observed in Kenya, Mozambique, and Uganda may be due to the issue that social constructs of the original MC-SDS are not quite as relevant to the social context in these countries. Since the aim of this paper was to assess the internal consistency of responses to the MC-SDS and the feasibility of its implementation in African countries, the analysis was limited to the process of adaptation and the measurement of reliability. Future qualitative research may shed more light on understanding cultural differences in social desirability and how it may influence the responses to the MC-SDS questionnaire in sub-Saharan African countries.

The study has several limitations. Although the tools had been translated and adapted for use in the local settings, it is possible that certain ideas were not understood the same way in these settings as they would be in the United States. The MC-SDS was designed for application in the United States and was based specifically on American ideas and expressions. Some of these are very specific and even though the questionnaire was adapted through a systematic process and some questions excluded, the use of this instrument in this context has not been validated. Though the results from this study show that the MC-SDS is a reliable instrument that can be delivered in an interview format, it is possible that the respondents misunderstood or interpreted the questions differently from its original design. Since this tool was intended to capture responses to cultural norms, developing a new survey modeled after the MC-SDS, but using questions that refer to local norms and practices, may be more effective at capturing SD.

Additionally, since these questionnaires were administered by an interviewer, it is possible that this face-to-face interview format may have enhanced the SD effect and influenced the respondents to reply in a more positive manner to conform to peer expectations. Lastly, due to monetary constraints, we were unable to implement a test-retest reliability procedure.

## Conclusion

The results of this study suggest that the MC-SDS can be effectively adapted and implemented in diverse settings in sub-Saharan Africa. The MC-SDS can be an important tool in measuring the effect of social desirability in survey responses based on self-reports used in current evaluations of HIV prevention programs, including those supported by PEPFAR. Formative assessments should be used to understand the various factors that may influence social norms of a community prior to adapting the MC-SDS instrument. Critical to the successful adaptation of the MC-SDS is the consultation with relevant stakeholders to ensure comprehension and cultural relevance of the MC-SDS statements.

## Competing interests

The authors declare that they have no competing interests. This study was supported by Samaritan's Purse with funding from the President's Emergency Plan for AIDS Relief. However, neither Samaritan's Purse nor PEPFAR had any role in the analysis of data or writing of the manuscript.

## Authors' contributions

AV conceived of the study, participated in the design of the study, collected data, participated in data analysis, and drafted the manuscript. NT participated in the formulation of the study, data collection and drafted the manuscript. KP drafted the manuscript. SA analyzed the data and participated in drafting the manuscript. All authors read and approved the final manuscript.

## Pre-publication history

The pre-publication history for this paper can be accessed here:

http://www.biomedcentral.com/1471-2288/11/162/prepub
